# Acute Hemorrhagic Encephalitis Responding to Combined Decompressive Craniectomy, Intravenous Immunoglobulin, and Corticosteroid Therapies: Association with Novel *RANBP2* Variant

**DOI:** 10.3389/fneur.2018.00130

**Published:** 2018-03-12

**Authors:** Abdulla Alawadhi, Christine Saint-Martin, Farhan Bhanji, Myriam Srour, Jeffrey Atkinson, Guillaume Sébire

**Affiliations:** ^1^Division of Pediatric Neurology, Department of Pediatrics, Montreal Children’s Hospital, Montreal, QC, Canada; ^2^Department of Medical Imaging, Montreal Children’s Hospital, McGill University, Montreal, QC, Canada; ^3^Pediatric Intensive Care Unit, Department of Pediatrics, Montreal Children’s Hospital, McGill University, Montreal, QC, Canada; ^4^Division of Neurosurgery, Department of Surgery, Montreal Children’s Hospital, McGill University, Montreal, QC, Canada

**Keywords:** Crohn disease, sclerosing cholangitis, sickle cell disease, Acute demyelinating encephalomyelitis, RANBP2, encephalitis, Acute necrotizing encephalopathy

## Abstract

**Background:**

Acute hemorrhagic encephalomyelitis (AHEM) is considered as a rare form of acute disseminated encephalomyelitis characterized by fulminant encephalopathy with hemorrhagic necrosis and most often fatal outcome.

**Objective:**

To report the association with Ran Binding Protein (*RANBP2)* gene variant and the response to decompressive craniectomy and high-dose intravenous methylprednisolone (IVMP) in life-threatening AHEM.

**Design:**

Single case study.

**Case report:**

A 6-year-old girl known to have sickle cell disease (SCD) presented an acquired demyelinating syndrome (ADS) with diplopia due to sudden unilateral fourth nerve palsy. She received five pulses of IVMP (30 mg/kg/day). Two weeks after steroid weaning, she developed right hemiplegia and coma. Brain magnetic resonance imaging showed a left frontal necrotico-hemorrhagic lesion and new multifocal areas of demyelination. She underwent decompressive craniotomy and evacuation of an ongoing left frontoparietal hemorrhage. Comprehensive investigations ruled out vascular and infectious process. The neurological deterioration stopped concomitantly with combined neurosurgical drainage of the hematoma, decompressive craniotomy, IVMP, and intravenous immunoglobulins (IVIG). She developed during the following months Crohn disease and sclerosing cholangitis. After 2-year follow-up, there was no new neurological manifestation. The patient still suffered right hemiplegia and aphasia, but was able to walk. Cognitive/behavioral abilities significantly recovered. A heterozygous novel rare missense variant (c.4993A>G, p.Lys1665Glu) was identified in *RANBP*2, a gene associated with acute necrotizing encephalopathy. RANBP2 is a protein playing an important role in the energy homeostasis of neuronal cells.

**Conclusion:**

In any ADS occurring in the context of SCD and/or autoimmune condition, we recommend to slowly wean steroids and to closely monitor the patient after weaning to quickly treat any recurrence of neurological symptom with IVMP. This case report, in addition to others, stresses the likely efficacy of combined craniotomy, IVIG, and IVMP treatments in AHEM. *RANBP2* mutations may sensitize the brain to inflammation and predispose to AHEM.

## Introduction

Acute hemorrhagic encephalomyelitis (AHEM) or acute hemorrhagic leukoencephalitis is considered a rare and extremely severe form of acute disseminated encephalomyelitis (ADEM). AHEM is characterized by an acute and rapidly progressive encephalopathy including hemorrhagic necrosis of the parenchyma of the central nervous system. It is usually fatal (1–3). Many treatment options have been used including intravenous (IV) steroids, intravenous immunoglobulins (IVIG), and plasmapheresis (4). There have been few reports of survival following early intervention with high-dose corticosteroid therapy and/or decompressive craniotomy (5–9).

RANBP2, a nuclear pore protein, has numerous roles in the cell cycle. RANBP2 is associated with microtubules and mitochondria suggesting roles in intracellular protein trafficking or energy maintenance and homeostasis of neuronal cells. RANBP2 mutations have been reported in acute necrotizing encephalopathy (ANE) which could present with coma, convulsions, and encephalopathy. The hallmark of ANE is multiple, symmetric brain lesions located in the thalami bilaterally, putamina, deep periventricular white matter, cerebellum, and brainstem. It could be triggered by a viral infection in previously healthy children (10).

We report a new case of AHEM associated to a Ran Binding Protein (*RANBP*)-2 variant and responsive to combined craniectomy, intravenous methylprednisolone (IVMP), and IVIG as inaugural manifestation of multisystemic autoimmunity in a girl with sickle cell disease (SCD).

## Case Report

**Figure 1:**
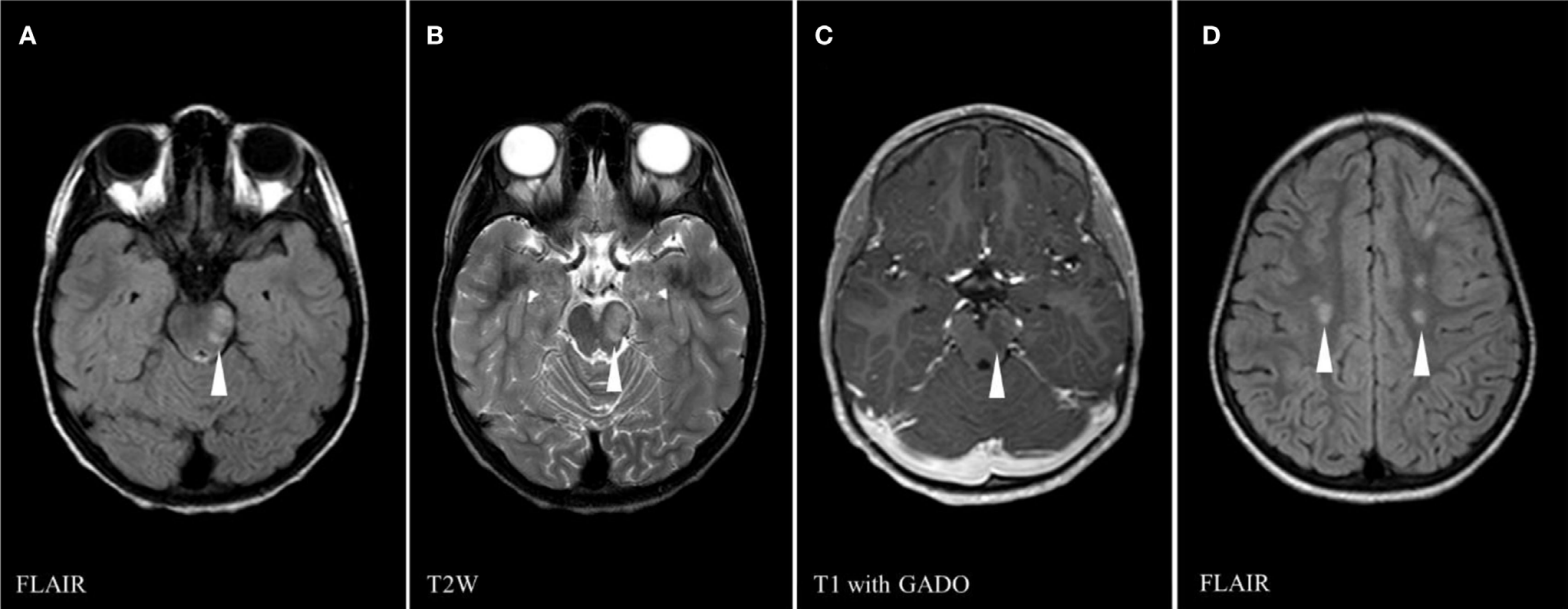
Brain magnetic resonance imaging on first presentation (day 5) showing multiple areas of abnormal signals (white arrowheads): left midbrain high signal on FLAIR sequence **(A)**, left midbrain hypersignal on T2 weighed (T2W) sequence **(B)**, central gadolinium enhancement of left midbrain lesion on T1 sequence **(C)**, and scattered supratentorial white matter foci of high FLAIR signal **(D)**.

A 6-year-old girl known for SCD treated on folic acid and hydroxyurea was admitted for new-onset diplopia [day 0 (D0): refers to the start of the diplopia] 6 weeks after respiratory tract infection due to rhinovirus. She was diagnosed with a fourth nerve palsy secondary to an acquired demyelinating syndrome. The initial brain magnetic resonance imaging (MRI) performed at D5 after onset of neurological symptom showed left midbrain and pontine edema with expansion of the brainstem, right caudate nucleus, and scattered supratentorial white matter foci of high T2/FLAIR signal (Figure 1). Brain MR angiography (MRA) showed a normal appearing circle of Willis. The cerebrospinal fluid (CSF) obtained by lumber puncture was normal (WBC 1 cells/μl, RBC 0 cells/μl, glucose 2.9 mmol/L, protein 0.18 g/L, and absent oligoclonal bands). The infectious workup including blood bacterial culture, CSF bacterial and viral cultures, nasopharyngeal aspirate (tested for Influenza A, Influenza B, Parainfluenza 1-2-3, Respiratory Syncytial Virus, Adenovirus, Coronavirus 229E, Coronavirus OC43, Metapneumovirus, Enterovirus, and Rhinovirus), and serologies for Epstein–Barr virus, *Mycoplasma pneumoniae*, HTLV I, HTLV II, HIV1, and Lyme disease were negative. Bartonella Henselae IgG was positive (1:1,280) reflecting a previously acquired common and self-limited infection in our area. Antinuclear antibodies (ANA) were positive (1:160). B12 and folate levels were normal. Smooth muscle antibodies were negative. Anti-mitochondrial antibodies were positive. Sedimentation rate was 65 mm/h. She was treated with five doses of IVMP (30 mg/kg/day) followed by 9 days of oral prednisone (1 mg/kg/day). At discharge, her neurological exam was significant only for vertical diplopia.

**Figure 2:**
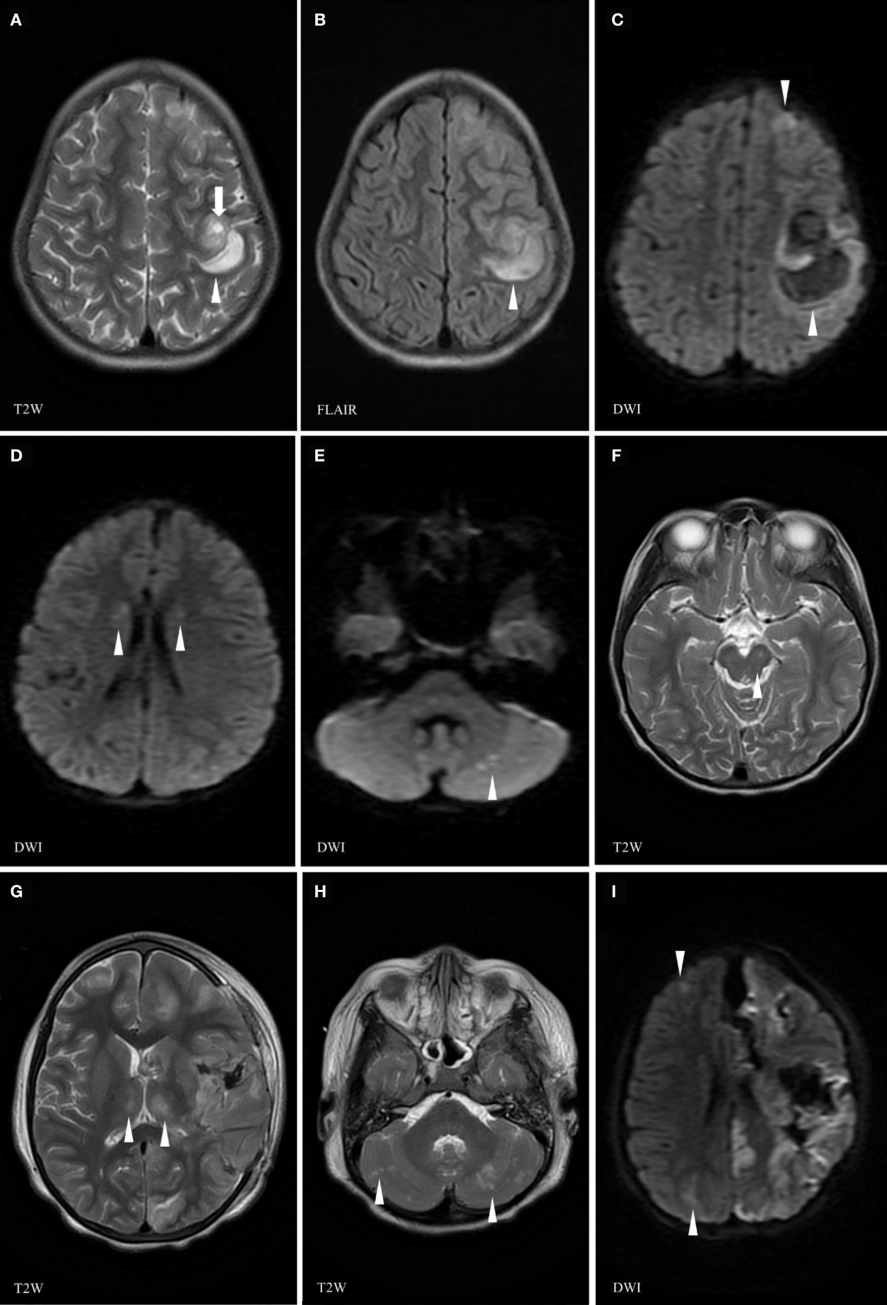
Brain magnetic resonance imaging (MRI) (day 30) at the time of the relapse of the neurological manifestations showing left frontoparietal cortical and subcortical areas of isointense T1, high T2 **(A)**, FLAIR **(B)**, and restricted diffusion **(C)** signals (arrowhead) increasing in size along the MRI examination, due to an active hyperacute bleed [arrow **(A)** pointing at spot sign indicating active bleed]. Area of high T2 and FLAIR signal changes (arrowheads) in the head of the caudate nucleus **(D)** and of abnormal diffusion weighed images (DWI) in the subcortical right cerebellar white matter (arrowheads) **(E)**. Previous left midbrain lesion has resolved with only a tiny residual of high FLAIR signal corresponding to minimal residual gliosis (arrowhead) **(F)**. Follow-up brain MRI at D33 showing a necrotico-hemorrhagic lesion in the left hemisphere **(G,I)**, interval evolution of cortical/subcortical lesions, with new lesions in the bilateral thalami **(G)**, cerebellar hemispheres **(H)**, and right cerebral hemisphere **(I)** (arrowheads indicate abnormal signals).

**Figure 3:**
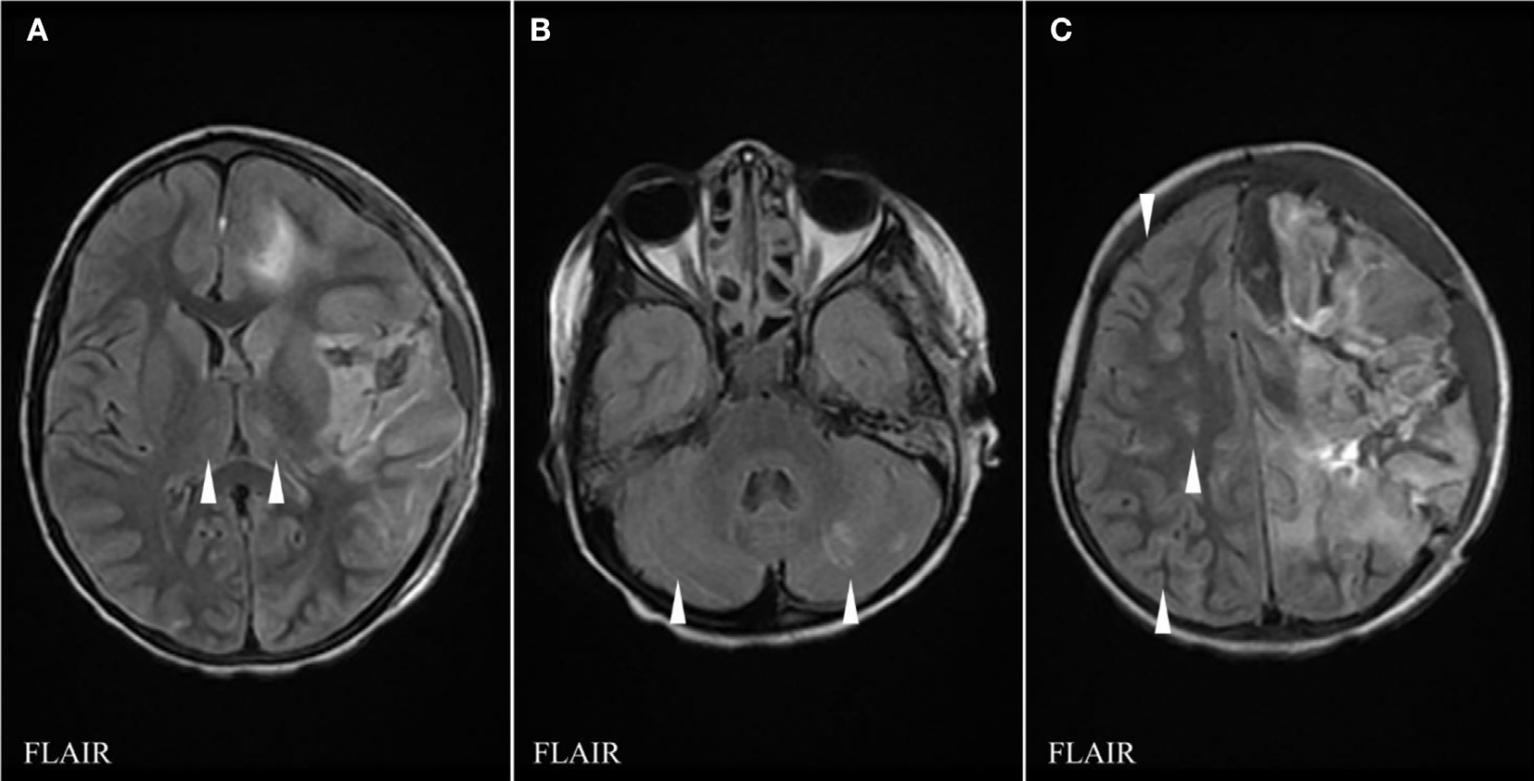
FLAIR sequences of brain magnetic resonance imaging (MRI) at day 39 showing interval evolution and improvement of thalamic **(A)**, cerebellar **(B)**, and right cerebral hemispheric lesions **(C)** with no additional lesions as compared to the previous MRI (arrowheads indicate hypersignal abnormalities).

She presented 1 month later with 5 days of upper respiratory tract infection symptoms, fever, headache, and a rapidly progressive right-hand weakness (D30) with normal alertness. She had normal blood pressure (120/81 mmHg). She was started on cefotaxime, vancomycin, and acyclovir. White cell count was 13.4 × 10^9^/L, hemoglobin was 7.8 g/L, and platelets were 239 × 10^9^/L. While in the MRI machine (D30) she deteriorated with vomiting and reduced level of consciousness (Glasgow Coma Scale dropped from 15 to 8 over 30 min). Brain MRI showed a rapid progression over a few sequences of an active bleed involving both superficial and deep gray matter as well as subcortical white matter of the left hemisphere anterior quadrant. Brain MRA was normal (Figures 2A–F). The patient was immediately brought out of the magnet and her physical exam demonstrated unequal dilated pupils. She received IV mannitol and hypertonic saline for the management of acute intracranial hypertension/herniation and was taken for surgery. She underwent left frontotemporoparietal decompressive craniotomy, evacuation of left frontoparietal intracerebral hemorrhage, and insertion of an external ventricular drain (EVD). Upon opening the skull, there was significant dural tension, and on opening the dura mater, there was a large amount of bleeding, in addition to brain swelling and necrosis. Estimated blood loss was 3.5 L. She received 8 units of packed red blood cells, 3 units of cryoprecipitate, 6 units of fresh frozen plasma, and 3 units of platelets. Coagulation profile showed international normalization ratio = 3.38, prothrombin time = 51.2 s, and partial thromboplastin time = 122 s. An intraventricular pressure monitor was inserted. She returned with stable vitals to PICU. At D31, the CT scan showed extensive multi-compartmental bleed involving the left frontoparietal lobes, the interhemispheric fissure, and the left hemispheric arachnoid spaces. New white matter lesions were detected in the left posterior parietal and occipital lobes and in the left caudate head. MRI at D33 showed interval worsening with disseminated gray and white matter non-hemorrhagic lesions in the right cerebral and both cerebellar hemispheres, bilateral deep gray nuclei, as well as new necrotic non-hemorrhagic lesions in the left hemisphere (Figures 2G–I). She was started on IVMP (30 mg/kg/day for 5 days) and IVIG (1 g/kg/day for 2 days). Repeat MRI at D9 showed no new parenchymal hemorrhage and partial resolution of the non-hemorrhagic lesions (Figure 3). Prednisolone was tapered course over 6 weeks. At discharge (D71), she was able to say a few words and had better power of her right side. Brain MRI performed 3 months later showed complete resolution of the non-hemorrhagic non-necrotic lesions, mainly seen in the right cerebral hemisphere and the cerebellum.

Brain biopsy of the hematoma, some small vessels, cortex, and white matter showed necrotic area, reactive and non-specific findings which could be entirely explained by compressive changes adjacent to a hematoma. There was diffuse microglial activation and signs of early microinfarcts. Blood, CSF and urine culture, and PCR (HSV1/2) were negative for bacteria and for viruses. CSF obtained through craniotomy and EVD performed at D32 showed elevated proteins 2.56 g/L, glucose 3.6 mmol/L, white blood cells 9 cells/μL, and red blood cells 1,341 cells/μL. ANA and anti-DNA antibody were negative.

Anti-extractable nuclear antigens (SSA-RO, SSB-LA, smith, RNP) were negative. Serum autoimmune antibodies panel (NMO, NMDAR, AMPA I/II, GAB, MAG, VGCC, MOG, YO, HU, RI) were negative but GAD antibody was slightly positive, possibly due to the IVIG infusion. EBV showed no signs of recent infection.

After discharge, the patient was started on regular transfusion exchange. Six months later, the patient was diagnosed to have Crohn’s disease and primary sclerosing cholangitis. Two years later, the patient still suffers right hemiparesis but is able to walk without support. She presents an expressive aphasia. Her intellectual abilities are average, or below the mean but in the normal range, except for the speed of information processing, verbal working memory, and some elaborated executive functions.

## Genetic Tests

A gene panel (Table 1) targeting inflammatory disorders and post-infectious necrotic encephalopathies found a heterozygous *RANBP2* missense mutation (NM_006267.4, c.4993A>G, p.Lys1665Glu). This mutation has not been previously reported in the HGMD database. This variant has been observed at a frequency of <0.01% across the entire Broad ExAC dataset of individuals without severe childhood onset disease (6/117,118 alleles). Analysis of amino acid conservation indicates that the wild-type amino acid Lys1665 is conserved in 59 of 60 mammals examined, including 12 of 12 primates, and in 25 of 34 non-mammalian vertebrates increasing the likelihood that a change at this position might not be tolerated. *In silico* tools predict that this variant is damaging (SIFT and Align GVGD).

**Table 1:** List of the 45 genes associated with acute hemorrhagic encephalomyelitis/autoimmune inflammatory disorders studied in our patient.

#	Gene abbreviation	Gene full name	OMIM #	#	Gene abbreviation	Gene full name	OMIM#
1	AP1S3	ADAPTOR-RELATED PROTEIN COMPLEX 1, SIGMA-3 SUBUNIT	615781	24	MYOM2	MYOMESIN 2	603509
2	AQP1	AQUAPORIN 1	107776	25	NLRC4	NLR FAMILY, CASPASE RECRUITMENT DOMAIN-CONTAINING 4	606831
3	AQP4	AQUAPORIN 4	600308	26	NLRP12	NLR FAMILY, PYRIN DOMAIN-CONTAINING 12	609648
4	CARD14	CASPASE RECRUITMENT DOMAIN-CONTAINING PROTEIN 14	607211	27	NLRP3	NLR FAMILY, PYRIN DOMAIN-CONTAINING 3	606416
5	CD8A	CD8 ANTIGEN, ALPHA POLYPEPTIDE	186910	28	NLRP7	NLR FAMILY, PYRIN DOMAIN-CONTAINING 7	609661
6	CECR1	CAT EYE SYNDROME CHROMOSOME REGION, CANDIDATE 1	607575	29	NOD2	NUCLEOTIDE-BINDING OLIGOMERIZATION DOMAIN PROTEIN 2	605956
7	ELANE	ELASTASE, NEUTROPHIL-EXPRESSED	130130	30	NRP1	NEUROPILIN 1	602069
8	FASLG	FAS LIGAND	134638	31	OCLN	OCCLUDIN	602876
9	FLT1	FMS-RELATED TYROSINE KINASE 1	165070	32	PDCD1	PROGRAMMED CELL DEATH 1	600244
10	HAX1	HCLS1-ASSOCIATED PROTEIN X1	605998	33	PLCG2	PHOSPHOLIPASE C, GAMMA-2	600220
11	IL10	INTERLEUKIN 10	124092	34	PSMB8	PROTEASOME SUBUNIT, BETA-TYPE, 8	177046
12	IL10RA	INTERLEUKIN 10 RECEPTOR, ALPHA	146933	35	PSTPIP1	PROLINE/SERINE/THREONINE PHOSPHATASE-INTERACTING PROTEIN 1	606347
13	IL1RN	INTERLEUKIN 1 RECEPTOR ANTAGONIST	147679	**36**	**RANBP2**	**RAN BINDING PROTEIN 2**	**601181**
14	IL2	INTERLEUKIN 2	147680	37	RBCK1	RANBP-TYPE AND C3HC4-TYPE ZINC FINGER-CONTAINING 1	610924
15	IL36RN	INTERLEUKIN 36 RECEPTOR ANTAGONIST	605507	38	RPS27A	RIBOSOMAL PROTEIN S27a	191343
16	IRF1	INTERFERON REGULATORY FACTOR 1	147575	39	SH3BP2	SH3 DOMAIN-BINDING PROTEIN 2	602104
17	ITGA4	INTEGRIN, ALPHA-4	192975	40	SLC29A3	SOLUTE CARRIER FAMILY 29 (NUCLEOSIDE TRANSPORTER), MEMBER 3	612373
18	KDR	KINASE INSERT DOMAIN RECEPTOR	191306	41	STAT1	SIGNAL TRANSDUCER AND ACTIVATOR OF TRANSCRIPTION 1	600555
19	LPIN2	LIPIN 2	605519	42	TMEM173	TRANSMEMBRANE PROTEIN 173	612374
20	MBP	MYELIN BASIC PROTEIN	15159430	43	TNFRSF11A	TUMOR NECROSIS FACTOR RECEPTOR SUPERFAMILY, MEMBER 11A	603499
21	MEFV	FAMILIAL MEDITERRANEAN FEVER GENE	608107	44	TNFRSF1A	TUMOR NECROSIS FACTOR RECEPTOR SUPERFAMILY, MEMBER 1A	191190
22	MVK	MEVALONATE KINASE	251170	45	VEGFA	VASCULAR ENDOTHELIAL GROWTH FACTOR A	192240
23	MX1	MYXOVIRUS RESISTANCE 1	147150				

*Bold characters indicate gene in which potentially pathogenic variant was detected*.

## Discussion

Several differential diagnoses of acute encephalopathy in a patient with sickle cell anemia can be considered. An infectious encephalitis, including herpes encephalitis, was ruled out by blood and CSF bacterial and viral cultures and negative HSV I/II PCR. Nasopharyngeal aspirate was negative for viruses. Some infections have been previously associated with necrotizing encephalitis such as Influenza A (11). SCD patients are prone to ischemic or hemorrhagic strokes (12). Primary hemorrhagic stroke is uncommon in pediatric SCD. Most cases were from adults and have been described in the context of previous ischemic stroke, aneurysms, low hemoglobin, acute chest syndrome, and hypertransfusions. Moreover, although hemorrhagic stroke has been described in SCD patients receiving transfusion or corticosteroids, it was in the context of elevated blood pressure which was not present in our case (13). This was ruled out as the MRI findings were not consistent with a specific vascular territory and normal arterial and venous flows were shown on vascular imaging. Another differential is posterior reversible encephalopathy syndrome which has been reported in SCD patients (13–16). However, it is unlikely in our case due to the severity of the brain injury and the absence of classic precipitating factors of posterior reversible encephalopathy syndrome such as high blood pressure. Macrophage activation syndrome could also lead to acute necrotic brain injury. However, it is associated to high ferritin and low triglycerides at the time of the encephalopathy, other multisystemic injuries, typical neuropathological findings, and recurrence over time, which were not noted in our patient (17). Parvovirus B19 has been described to cause encephalopathy in sickle cell patients. It is associated with aplastic anemia. It caused punctate areas of hemorrhages in the basal ganglia, periventricular white matter, and mainly along the posterior parietal cortex. This was attributed to parvovirus B19-induced vasculitis (18). In our patient, there was no sign of aplasia or any neuroradiological finding of parvovirus B19 infection. Finally, acute encephalitis has been observed in SCD patients in the context of arterial hypoxemia from fat embolism, pulmonary embolism, sudden anemia, or acute chest syndrome due to pneumonia (19). This was ruled out as the patient did not have clinical or radiological signs of acute chest syndrome or embolism and there was no arterial hypoxemia.

Acute hemorrhagic encephalomyelitis has been described in pediatric patients following ADEM or ADEM-like episodes (20, 21). AHEM is the most plausible diagnosis in our patients based on the clinical and radiological presentation, the preceding ADEM-like episode, and the exclusion of other etiologies of acute encephalopathy. Other patients with AHEM have been described in the SCD context (7, 19). Many treatment options have been used to treat AHEM; of these, IV steroids have been associated with survival following aggressive, high-dose corticosteroid therapy (5–9, 22–25).

Autosomal dominant mutations (with incomplete penetrance) in *RANBP2* have been associated with susceptibility to infection-induced necrotizing encephalopathy (26, 27). Previously healthy patients with pathogenic mutations in *RANBP2* can present acutely with encephalopathy and convulsions in the context of an infection, with brain imaging revealing involvement of the brainstem, thalami, putamina, cerebellum and external capsules, and claustrum (10). Our patient has a similar presentation and imaging features as infection-induced necrotizing encephalopathy, including bilateral thalamic involvement. The rare heterozygous previously unreported variant we identified in *RANBP2* affects a very conserved aminoacid and is predicted deleterious using *in silico* tools (a prediction tool performing a fast bioinformatics analysis which can predict the pathogenicity of a variant based on the change to an amino acid). It is possible that this variant is pathogenic and responsible for the clinical phenotype. There is an overlap between the diagnostic criteria of AHEM and those of acute hemorrhagic encephalopathy (25, 26) making possible that both entities might be part of the same pathophysiological continuum. *RANBP2* is a protein playing an important role in the energy homeostasis of neuronal cells (28). Hence, *RANBP2* dysfunction might make neuronal cells much vulnerable to energy failure and necrosis when exposed to inflammatory or other stresses, such as those implicated in AHEM.

## Ethics Statement

This study was carried out in accordance with the recommendations of our institutional ethic committee. Written informed consent was obtained from all the participants for the publication.

## Author Contributions

All authors participated in gathering the data, designing the article, and discussing and editing the manuscript.

## Conflict of Interest Statement

There is no conflict of interest. This manuscript has been seen and approved by all co-authors. All the authors fulfill the authorship credit requirements. AA wrote the first draft of this manuscript. No honorarium grant or other form of payment was received for the preparation of this manuscript.
